# Hematometra within a cesarean scar defect in a perimenopausal woman: A case report

**DOI:** 10.1111/jog.16253

**Published:** 2025-02-23

**Authors:** Atsushi Murakami, Shunichiro Tsuji, Yu Koikawa, Mari Nakata, Suzuko Moritani, Takashi Murakami

**Affiliations:** ^1^ Department of Obstetrics and Gynecology Shiga University of Medical Science Otsu Shiga Japan; ^2^ Department of Obstetrics and Gynecology National Hospital Organization Higashiomi General Medical Center Higashi‐ohmi Shiga Japan; ^3^ Department of Clinical Laboratory Medicine Shiga University of Medical Science Otsu Shiga Japan

**Keywords:** acute abdominal pain, cesarean scar defect, endometriosis, hematometra, perimenopause

## Abstract

Cesarean scar defect (CSD) is a long‐term complication of cesarean section (CS). However, its risks for perimenopausal women remain unclear. We present a rare case of CSD involving hematometra in a perimenopausal woman, which led to emergency hysterectomy. A 51‐year‐old woman with a history of CS presented with acute abdominal pain. Transvaginal ultrasonography and magnetic resonance imaging identified a hematometra within a large CSD extending into the cervical myometrium. Emergent hysterectomy was performed due to persistent pain. Histopathology revealed a fresh hematoma within the CSD, with ectopic endometrial glands and hemorrhage on its walls. This case highlights the pathogenesis of hematometra within a CSD and underscores its potential to cause acute abdomen in perimenopausal women.

## INTRODUCTION

Cesarean section (CS) is a life‐saving procedure for both mothers and fetuses. The global frequency of CS has risen significantly, with 29.7 million births by CS in 2015, nearly double the number in 2000.^1^, ^2^ Consequently, cesarean scar defect (CSD), a long‐term complication of CS, has become a concern. CSD is defined as a thinning myometrial space with a depth of at least 2 mm,^3^, ^4^ with a prevalence ranging from 24% to 84%.^1^ CSD can cause various gynecological symptoms, including postmenstrual spotting, dysmenorrhea, and chronic pelvic pain^1^, ^5^, ^6^; the prevalence of these symptoms among women with CSD is 63.8%, 53.1%, and 39.6%.^7^ In addition, CSD can cause secondary infertility,^1^, ^8^ although the exact prevalence is unclear. While surgical repair of CSD is well documented for improving fertility,^1^, ^5^ little is known about the risks of CSD for perimenopausal women not seeking conception.

We report a rare case of hematometra within a CSD in a perimenopausal woman, necessitating a hysterectomy.

## CASE PRESENTATION

A 51‐year‐old woman with a history of CS due to twin pregnancy 12 years ago presented with acute lower abdominal pain on the first day of menstruation. She had a history of postmenstrual spotting since the CS, along with hypomenorrhea and abnormal genital bleeding in the past year. In addition, she had been amenorrheic since her last menstruation 3 months prior to the current one. She had not experienced dysmenorrhea until this emergency visit, during which she reported her worst menstrual pain to date.

**FIGURE 1:**
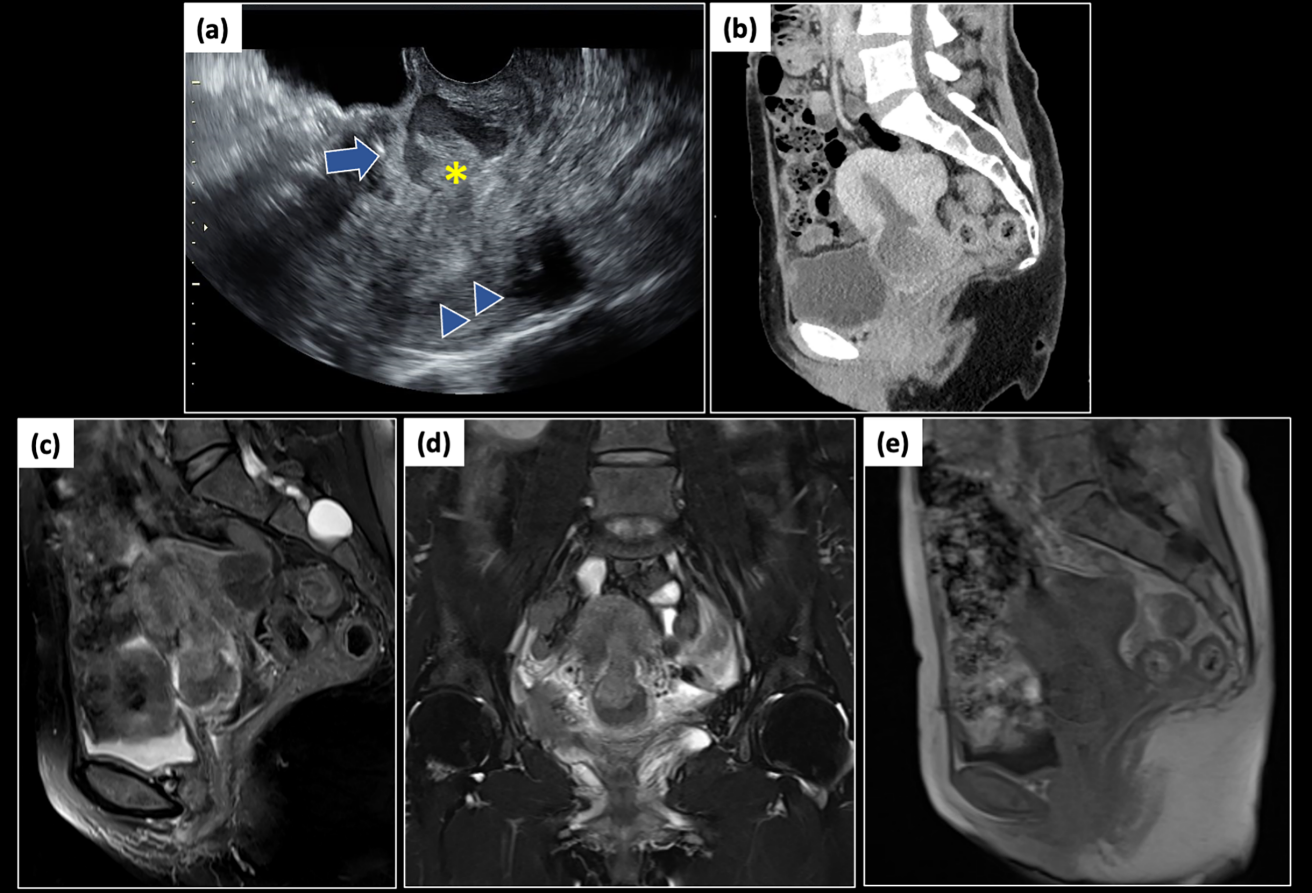
Imaging findings of hematometra within cesarean scar defect. (a) Transvaginal ultrasonography revealed the existence of hematometra within CSD and high‐echoic objects flowing back and forth between the uterine cavity and CSD. The arrow indicates CSD, the triangles indicate the uterine cavity, and the asterisk indicates high‐echoic objects. (b) CT scan revealed no evidence of extravasation surrounding the CSD. (c, d) MRI on T2WI: (c) a sagittal section; (d) a coronal section. CSD was hypointense, and the blood flowing from the uterine cavity into it was hyperintense. (e) MRI on T1WI of sagittal section. Both the CSD and the blood flowing into it were slightly hypointense. CSD, cesarean scar defect; CT, contrast‐enhanced computed tomography; MRI, magnetic resonance imaging; T2WI, T2‐weighted images; T1WI, T1‐weighted images.

A vaginal examination revealed a small amount of abnormal uterine bleeding and age‐related cervicovaginal atrophy. Transvaginal ultrasonography showed severe CSD with fluid retention extending into the uterine cavity (Figure 1a). High‐echoic objects, likely minor hematomas, were detected in the CSD and moved between it and the uterine cavity without being expelled from the cervix (Figure 1a). Pressure pain on the CSD was observed during the transvaginal probe examination. Contrast‐enhanced CT revealed no extravasation around the CSD or other causes of acute abdomen (Figure 1b). MRI was performed for qualitative evaluation. Fat‐suppressed T2‐weighted images (T2WI) showed the CSD as hypointense and the blood flowing into it as hyperintense (Figure 1c,d). On T1‐weighted images (T1WI), both the CSD and the blood were slightly hypointense (Figure 1e). In addition, the CT and MRI findings also suggested another possibility that a malignant uterine mass had formed a hematometra. She was afebrile, and her vital signs were stable. Blood tests showed elevated white blood cells (14 800/μL) and C‐reactive protein (0.84 mg/dL) without anemia, coagulation abnormalities, or organ damage. A pregnancy test was negative. Uterine cervical cytology was negative for intraepithelial lesion or malignancy. Culture of vaginal discharge and polymerase chain reaction tests for *Neisseria gonorrhoeae* and *Chlamydia trachomatis* were also negative.

**FIGURE 2:**
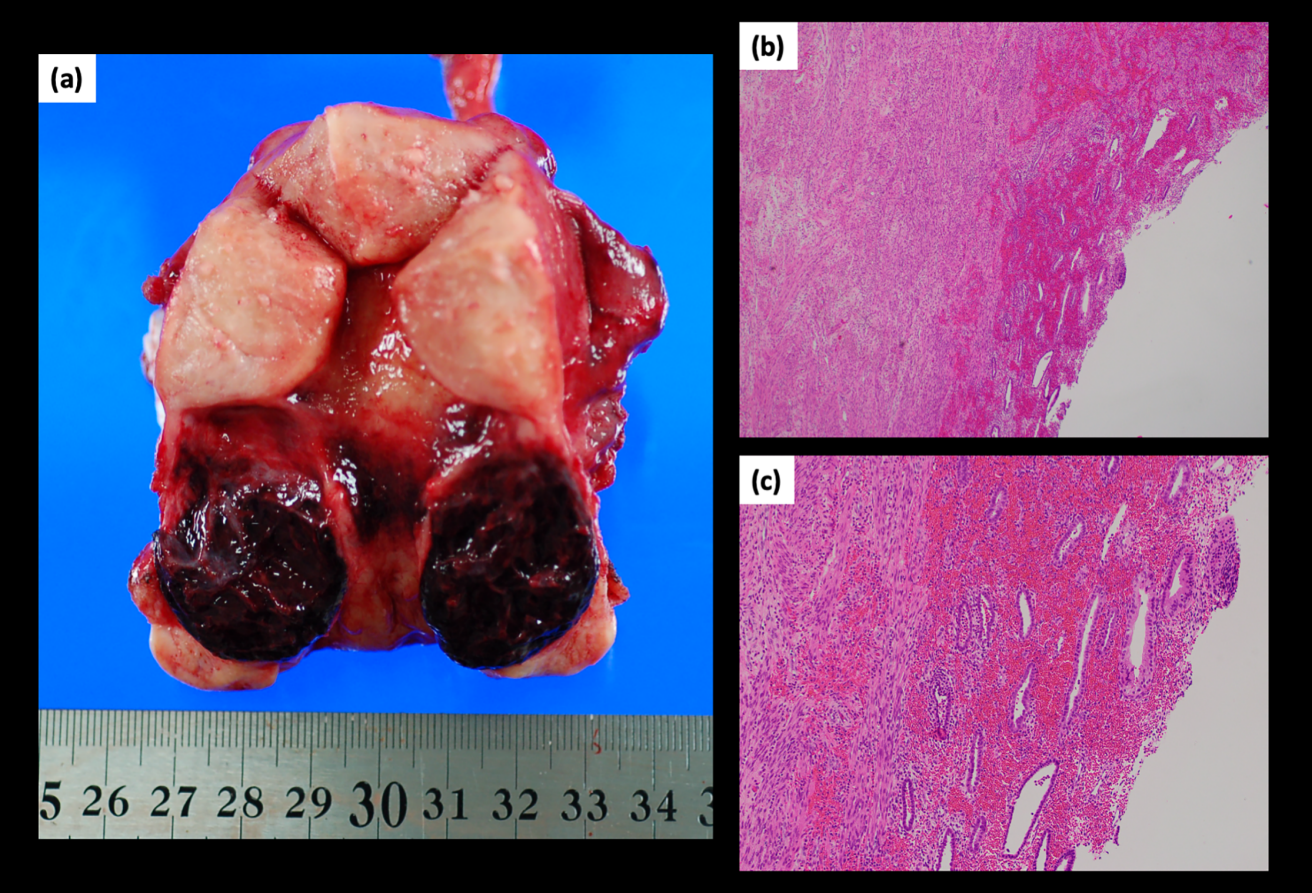
Extraction specimen and histopathology findings. (a) The excised specimen showed that a relatively fresh hematoma filled the CSD, extending into the interior of the cervical myometrium. (b, c) Histologic examination revealed that the walls of the hematoma were covered with ectopic endometrial glands with hemorrhage. Hematoxylin–eosin stain; original magnification: ×40 (b), ×100 (c). CSD, cesarean scar defect.

The patient's condition was diagnosed as a massive hematometra in the CSD. Due to persistent abdominal pain and concerns about uterine rupture or infection in the CSD area, an emergency hysterectomy was performed. The excised specimen revealed a fresh hematoma filling the CSD and extending into the cervical myometrium (Figure 2a). Upon insertion of a sonde into the uterus, a constriction was noted at the internal uterine ostium. Histologic examination with hematoxylin–eosin staining showed that the hematoma walls were lined with ectopic endometrial glands and hemorrhage (Figure 2b,c), indicating endometriosis. No intraoperative or postoperative complications occurred.

## DISCUSSION

We presented a rare case of hematometra within a CSD in a perimenopausal woman. This case suggests that impaired menstrual blood drainage due to cervical stenosis, associated with the presence of CSD and endometriosis‐related bleeding in the CSD region, led to the rapid development of hematometra.

Similar to the current case, Chin et al. reported a case of bleeding in a postmenopausal woman with CSD.^9^ Both cases share similarities, with acute abdomen, hematometra formation within the CSD, and hysterectomy as the chosen treatment. However, the previous case differed in that the patient had no premenopausal symptoms, such as postmenstrual spotting or irregular genital bleeding; the hysterectomy was performed on a standby basis; and no evidence of endometriosis was found in the CSD. These differences may explain the rapid hematometra growth in this case, necessitating emergency hysterectomy.

Acquired causes of hematometra typically include cervical stenosis from atrophy, radiotherapy, conization, or cervical neoplastic lesions.^10^, ^11^ In this case, the hematometra extended into the cervical myometrium, resembling a uterine cervical tumor. Age‐related cervicovaginal atrophy likely contributed to impaired menstrual blood expulsion, creating a cycle of cervical stenosis, menstrual obstruction, and increased blood inflow into the CSD. The patient's hypomenorrhea and irregular genital bleeding over the past year may have been early signs of gradually increasing fluid in the CSD, culminating in hematometra. Additionally, pathological evidence of endometriosis in the CSD suggests that ectopic bleeding exacerbated the condition. Previous studies have reported ectopic endometrium development around the CSD and hysteroscopic observations of bleeding from the CSD myometrium,^8^, ^12^ supporting this hypothesis.

MRI findings aligned with acute hemorrhagic changes: slightly hypointense hematometra on T1WI and very hypointense hematometra with slightly hyperintense menstrual blood in the CSD on T2WI, consistent with the acute phase (1–3 days).^13^ This, coupled with acute abdominal pain without prior dysmenorrhea, supports the acute nature of this case.

No established prevention or treatment exists for hematometra within a CSD. Given the ectopic endometrium surrounding the hematoma, general endometriosis treatments, such as progestin‐based therapies, may help prevent similar cases. However, identifying high‐risk patients remains a challenge, requiring further research.

There are four potential treatment options: (1) expect spontaneous evacuation, (2) transvaginal drainage with or without hysteroscopy, (3) attempt evacuation via surgery, or (4) perform a hysterectomy. Option (1) is the least invasive but unsuitable for patients with severe pain and may increase the infection risk due to prolonged blood retention. Warrier et al. reported a CSD abscess refractory to antibiotics, ultimately requiring hysterectomy,^14^ indicating that (1) may not be viable in such cases. Option (2) includes uterine preservation therapy, which facilitates drainage via cervical dilation. Further alternatives may include hysteroscopic observation and hemostasis in CSD. Unfortunately, in this case, the cervix was so narrowed by stenosis that a sonde could not pass through, making cervical dilation difficult. Even if slight cervical dilatation were possible, it would have resulted in inadequate drainage and hematometra re‐aggravation. Therefore, option (2) was not selected. Option (3) also preserves the uterus, but it is more invasive. In addition to observation and hemostasis by hysteroscopy, laparoscopic or abdominal surgery for removing and repairing the defective myometrium is envisioned. However, this case would require removing most of the myometrium due to hematoma expansion, creating a larger defect, and risking secondary complications like recurrent hematoma, uterine rupture, irregular bleeding, or abscess. Since the patient was perimenopausal and did not desire uterine preservation, option (3) was not chosen. Option (4), while radical, was deemed appropriate due to her persistent pain, concerns about complications from operative drainage, and lack of interest in fertility preservation. In addition, there are cases of gynecologic cancers in women with hematometra. So, regarding making a decision on the treatment, hysterectomy also included diagnostic meaning to exclude gynecologic malignancy, if the cervix was too narrow to perform intrauterine histological evaluation.

In conclusion, perimenopausal women with CSD are at risk for acute abdominal pain from hematometra development. Preventative strategies and effective treatments remain undefined, warranting further research.

## AUTHOR CONTRIBUTIONS

**Atsushi Murakami:** Conceptualization; data curation; investigation; methodology; project administration; resources; validation; visualization; writing – original draft; writing – review and editing. **Shunichiro Tsuji:** Conceptualization; project administration; supervision; writing – review and editing. **Yu Koikawa:** Data curation; investigation; resources; visualization. **Mari Nakata:** Investigation; resources; writing – review and editing. **Suzuko Moritani:** Investigation; resources; supervision; visualization; writing – review and editing. **Takashi Murakami:** Supervision; writing – review and editing.

## CONFLICT OF INTEREST STATEMENT

The authors declare no conflict of interests for this article.

## ETHICS STATEMENT

Informed consent was obtained from the patient to publish data and images.

## Data Availability

Data sharing is not applicable to this article as no datasets were generated or analyzed during the current study.
